# The socioeconomic factors of investment-induced petroleum consumption: case of fast developing Chinese economy

**DOI:** 10.1007/s13202-022-01518-w

**Published:** 2022-06-10

**Authors:** Muhammad Jawad Sajid, Zhang Yu, Syed Abdul Rehman

**Affiliations:** 1 School of Engineering Management, Xuzhou University of Technology, Xuzhou, 221006 Jiangsu China; 2 Department of Business Administration, ILMA University, Karachi, 75190 Pakistan

**Keywords:** Economy, Embedded environmental impact, Fossil fuel, Input–output analysis (IoA), Socioeconomic factor, Structural decomposition analysis (SDA), Sustainability

## Abstract

Capital investment stimulates a sizable portion of petroleum consumption, especially in emerging economies. However, investment-embedded petroleum consumption (IEPC) and the socioeconomic factors that influence it are not well studied. Our study's objective is to close this research gap. Our article estimates the effects of petroleum intensity, technology, investment structure, and economic development on China's IEPC using input–output and bipolar structural decomposition analysis. Additionally, our article develops a previously mostly unknown index of investment intensity. The findings indicated that, on average, between 1990 and 2016, investment induced nearly 30% of China's total final demand-embedded petroleum consumption. On average, petroleum intensity had the most significant decreasing effect on the Chinese IEPC. Averagely, technology had a positive impact, but from 2010 to 2016, it had a noticeable negative impact (− 1.51 exajoule). Both investment intensity and economic development had a significant upward effect. The impact of investment intensity was the smallest of all the factors. Disaggregation of the effects of socio-economic factors at the sectoral level revealed distinct patterns. Thus, by focusing on the socioeconomic dynamics of key sectors, the factors' current decreasing effects can be maximized, and their increasing effects minimized.

## Introduction

Globally petroleum (oil) is the topmost consumed fossil fuel. In 2020 petroleum accounted for approximately 38% of worldwide fossil fuel consumption [based on (Ritchie and Roser 2020a)]. It accounts for 90% of global transportation needs (WWF-World Wide Fund For Nature 2020). Petroleum facilitated the development and distribution of a wide range of consumer goods, revolutionized travel, and improved people's quality of life (WWF-World Wide Fund For Nature 2020). Compared to other main fossil fuel types, coal and gas's 114 and 52.8 years of reserves left, the petroleum only has 50.7 years of reserves left (Ritchie and Roser 2020b). Therefore, to increase the timeframe of the petroleum reserves availability and reduce environmental pressures besides switching to alternative and renewable energy sources, it is also vital that the current petroleum consumption patterns are improved. In particular, the mitigation of petroleum consumption embedded in capital investments^1^ that compromises a significant portion of global gross domestic product [almost 25.15% of global GDP in 2020 (World Bank national accounts data 2022)] and is a final demand trait of nearly all kinds of primary, secondary and tertiary industries in both the developed and developing economies can help achieve this task efficiently and effectively.

The petroleum sector is critical to propelling China's economic growth (Chen et al. 2020). Currently, China is the world's second-largest petroleum consumer (Worldometer 2016a). China is the world's fourth-largest petroleum producer, and the country is ranked in 14th place in terms of petroleum reserves (Worldometer 2016a). Unlike the USA, which is the world's largest producer and consumer of petroleum (U.S. Energy Information Administration 2021a) and a net annual petroleum exporter in 2020 (U.S. Energy Information Administration 2021b)—China imports petroleum to meet 59% of its needs (Worldometer 2016a). In fact, without imports, current Chinese petroleum consumption can deplete China's petroleum reserves in merely five years (Worldometer 2016a). In 2020 COVID-19 reduced the global petroleum consumption significantly, including significant reductions in the USA, European Union, and India (bp (British petroleum) 2021). China was among the few nations that saw a considerable increase of 1.6% in petroleum consumption (bp (British petroleum) 2021).

Furthermore, total capital investment in China in 2020 was around 43.1% of GDP, which was much higher than in other nations throughout the globe (Textor 2021). Because petroleum products are used in nearly every industry, China's socioeconomic growth requires a steady supply of petroleum resources (Xu et al. 2011). Given the excessive reliance of China on imported petroleum, insufficient local petroleum deposits, current COVID-19, other supply chain disruptions (trade disputes, natural and weather risks, security, wars, etc.), and extremely high levels of capital investments make China a vital frontier for the analysis of IEPC and its drivers.^2^ By IEPC, we mean the fraction of petroleum consumed (used) by industries (sectors) of target regional, national, or global economies to provide products or services for the development (production) of fixed capital assets. Meanwhile, the drivers of IEPC provide information on the various socioeconomic factors that have historically influenced the growth of IEPC in an economy or group of economies (both positively and negatively).

Given the importance of Chinese fossil fuel consumption and fixed capital investment, numerous previous studies have looked into the environmental aspects of these two domains. Particular attention has been paid to the fossil fuels embedded in China's inter-industrial fossil fuel chains (Sun et al. 2016; Sajid et al. 2022) and final demand (Fu et al. 2021). Furthermore, some studies have examined the environmental consequences of China's capital investment (Cao et al. 2018, 2019a, b; Södersten et al. 2018a; Sajid et al. 2021a). However, the following research gaps in the related literature remain unfilled. First, associated studies rarely consider investment-embedded industrial (sectoral) environmental impacts. Second, the socioeconomic factors of investment-embedded fossil fuel consumption, such as petroleum, are understudied. Third, studies such as Södersten et al. (2018a) that looked at investment-embedded environmental impacts (in this case, carbon emissions) only looked at input–output multipliers and investment structure. Fourth, final demand-embedded environmental impact studies have generally not considered any factor representing the effect of investment intensity on embedded environmental impacts.

Our study makes to following contributions to the body of related knowledge. Firstly, we have estimated the temporal volume and factors of China's IEPC using the input–output and bipolar SDA methods. Secondly, by considering petroleum, which is a vital fossil-fuel category, we overcome the overall scarcity of literature on the factors of investment-embedded fossil fuel consumption. Thirdly, compared to previous research considering only two factors of IEPC, we in this study have investigated the impacts of petroleum intensity, technology (Leontief effect), investment structure, investment intensity, and economic development. Finally, the index of investment intensity that estimates the investment growth compared to GDP is introduced in our study. By presenting the effects of investment compared to total temporal gross production capacity (GDP), this ratio serves as a proxy for utilizing income (GDP)^3^ on investment compared to consumption. In other words, national choice of making investments instead of consuming. A positive impact indicates the share of investment has increased in temporal GDP growth, resulting in a decreased percentage of final consumption. In contrast, a negative effect will indicate the contrary.

Our research can help reduce petroleum demand by estimating the IEPC of the world's second-largest petroleum consumer. Given the current insufficient local deposits to meet consumption (Worldometer 2016a), the high investment ratio in Chinese GDP (Textor 2021), and China's leading position as both a producer and consumer of global carbon emissions (Sajid et al. 2021a), this has significant economic, political, and environmental benefits. Aside from China, our work indicating the key factors of IEPC can be beneficial in general for other Asian, African, and Latin American developing economies, particularly large oil importers such as India, South Africa, and Mexico, to effectively plan environmentally conscious fixed investments. This, in turn, can provide both economic and environmental benefits by reducing reliance on imported petroleum (i.e., decreasing trade deficit) and greening future investments in fixed assets.

## Literature review

The literature review section presents some pertinent literature on final demand-embedded environmental impacts. The section also depicts relevant literature on the decomposition of factors of final demand-embedded environmental impacts.

### Literature review on the final demand-embedded environmental impacts

In recent years, there has been a lot of focus on the final demand-embedded environmental impacts. Under these studies, all environmental impacts incurred during product production are allocated to final consumers (household and government), trade (imports or exports), and investment (GFCF, Inventory Changes, and Acquisitions (less disposals) of valuables) (Cao et al. 2018; Sajid et al. 2021a). Leontief's input–output model (IoT) has traditionally been used to estimate the environmental impacts of various final demand categories. Two main accounting methods have emerged for quantifying national final demand-embedded environmental impacts: production-based accounting (PBA) and consumption-based accounting (CBA). Because the PBA method considers the entire national direct environmental impacts, exports rather than imports are included alongside other final demands. While the CBA approach considers national consumption, imports are regarded as a representative of final trade. However, whether the PBA or CBA is used, capital investment is included as a final demand category in both approaches.

When we examine the literature on environmental impacts embedded in final demand, we discover that pollutant, carbon, materials, and energy use impacts are typically embedded in final demand. For example, Oita et al. (2016) quantified the nitrogen pollution embedded in international trade. Lenzen et al. (2021) calculated the global material footprint embedded in a country's final demand. Tian et al. (2014) estimated the final demand induced by China's regional PBA and CBA carbon footprints. Mi et al. (2016, 2019) quantified the final demand-stimulated CBA emissions for Chinese cities. Meng et al. (2017) investigated the final demand-embodied black carbon CBA emissions for China's major cities. Sajid et al. (2020) calculated the intermediate sectoral consumption-embedded emissions of Chinese rural and urban households using a consumption–consumption method. Fan et al. (2016) estimated the PBA and CBA carbon emissions of 14 major countries. Cortés-Borda et al. (2015) quantified the solar energy embedded in international trade. Fu et al. (2021) studied the energy embedded in the trade between countries of Belt and Road (BRI). Pan et al. (2021) compared the importance of the intensity vector in accounting for embodied energy. Sun et al. (2016) investigated the embedded energy transfer between different sectors of China. Sajid et al. (2022) quantified the intermediate sectoral supply-chain-embedded coal, petroleum, and natural gas linkages. Chen et al. (2019a) concluded, based on a review of 2608 web of science core collection articles, that embodied energy can provide a more holistic view of energy use and demand and a better scientific basis for policymaking to improve energy security.

In particular, the literature on the embodied impacts of capital investment has sometimes considered the investment as an intermediate production industry (capital formation endogenization) and embedding the environmental effects into the investment. Cao et al. (2018) estimated the Chinese final demand-embedded CBA emissions by considering the investment as a production sector. Sajid et al. (2021a, b, c), considering the capital investment and exports as intermediate sectors, estimated the Chinese consumers' PBA emissions. Chen et al. (2018) calculated the global CBA carbon emissions by endogenizing the capital investment. Södersten et al. (2018b) also estimated the global CBA greenhouse gas emissions after endogenizing fixed capital as a production sector.

### Literature review on factors impact decomposition

The SDA, index decomposition (IDA), structural path (SPA), and sensitivity (SA) analyses are commonly used with the input–output model in the related literature to decompose the effects of different factors on final demand-embedded environmental impacts. We present some of the pertinent literature on this topic in the following sections.

Li et al. (2020) used the SPA and SDA methods to estimate the impact of energy intensity, input structure, and final demand on Chinese embodied energy intensities. As per their findings, the final demand category of capital investment was primarily responsible for the positive effects on embedded energy intensity between 2012 and 2017. Meanwhile, the intensity factor embedded the most considerable reducing impact. Yan and Su (2020), using additive and multiplicative spatial SDAs, studied the energy intensity, Leontief structure (technology), final demand category, and final demand structure effects on the energy performance of China's major cities of Beijing, Shanghai, Tianjin, and Chongqing. Domestic investment, energy intensity, and Leontief effects played a considerable role in energy performance. Liu et al. (2019) employed IDA analysis to investigate the economic activity (growth), Leontief, and structural effects of energy consumption and intensity in Chinese regions. Their decomposition findings showed that economic growth drove energy consumption, but the energy intensity effect dampened overall consumption growth. While the structural impact was detrimental, it had little impact on rising energy use. Huang et al. (2022) assessed the impact of energy intensity, per capita consumption, production structure, population size, and consumption structure on China's provincial CBA-related energy consumption. The authors accomplished this goal by combining spatial independent variable lag modeling and SDA techniques. According to their findings, energy intensity and production structure played a significant role in Chinese energy saving. The population growth and consumption per capita had a growing effect. There was no discernible effect of consumption structure on energy consumption in comparison. Zhang et al. (2017a) evaluated China's whole primary energy supply chain using the SPA approach. Not only did their findings indicated that capital investment accounted for the lion's share (49.5%) of primary energy consumption, but also that the most important primary energy path began with investment and ended with "Coal Mining and Washing." Supasa et al. (2016) used the SDA approach to quantify the effects of energy efficiency, production structure, consumption structure, final demand category, and final consumption level on various kinds of fossil fuel use in Thailand. The findings indicated that the final demand impact was the most influential element in determining the effectiveness of energy conservation, but the energy efficiency effect was not as successful in reducing energy consumption as predicted.

Some studies have also considered the drivers of specific fossil fuel types. For example, Wang and Yang (2020) estimated the effects of final demand and its scale effect, oil intensity, production structure, nationality structure, industrial correlation effect, and intermediate product export and import trade on Germany's oil footprint using the SDA method. Their decomposition study revealed that the scale impact of final demand was the primary driver driving the development of oil footprints in Germany's international trade, more than offsetting the decline in oil consumption intensity. The influence of production structure was critical in the embodied oil trade, and Germany's low-oil-intensive industrial structure was the primary cause of Germany's net embodied oil imports. Wang and Song (2019) assessed India's coal footprint's PBA and CBA drivers using the SDA technique. The authors considered the effects of coal intensity, intermediate product requirements, and domestic and international final demand. Their findings indicated that India's coal intensity was the primary driver of its increased coal use, followed by final domestic demand. Andreoni (2022) investigated the impact of coal intensity, living standards, and population on regional coal consumption in China using the IDA technique. According to their findings, the primary driver of increased coal use was economic prosperity. Coal intensity aided in containing the rise in coal consumption in the most developed regions. Regional differences in internal migration rates significantly impacted the population effect.

Apart from the drivers of energy consumption and the various types of energy, considerable research has been conducted on energy-related carbon emissions and intensities. Using the complete average decomposition analysis, Kang et al. (2022) investigated how the intensity of black carbon emissions, intermediate input structural adjustment, final local demand, inter-provincial trade, and global exports affect Chinese black carbon emissions. According to the findings, the intensity of black carbon emissions significantly reduced emissions across all provinces. Between 2007 and 2012, the impact of structural adjustment on intermediate inputs was negative. The impact of intermediate input structure on black carbon emissions shifted from negative to positive as the tertiary industry became the primary driver of economic development, demonstrating that current industrial structure adjustments were not conducive to black carbon emission reduction. Sajid (2021) used the SDA and regional sensitivity analysis to investigate the effects of consumption structure, emission intensity, consumption tendency, income per capita, Leontief effect, population structure, and population on embedded industrial consumption emissions in Chinese households (DEIC). According to the findings, income per capita was the fundamental driver of DEIC emissions growth for rural and urban families. The consumer sector's Leontief effect (technology) had the second greatest impact on rural and urban house DEIC emissions. The second most sensitive variable, on the other hand, differed between rural and urban families. The emission intensity of rural households and the consumption proclivity of urban families had the most significant negative impact on their respective DEIC emissions.

Sajid (2020) used the SDA method to investigate the effects of household consumption structure, emissions intensity, population, production technology, propensity to consume, and per capita income on Pakistani household demand-embedded carbon emissions. According to the study's findings, rising household final demand-embedded carbon emissions were primarily driven by consumption proclivity, population, and per capita income from 2005 to 2015. Emission intensity had the greatest negative impact on emissions. Chen et al. (2019b) used the SDA to investigate the impacts of energy intensity, energy-carbon emissions, Leontief, size, and structure on the embedded carbon emissions in Chinese regional commerce. Their findings suggest that scale impact was the primary driver of embodied carbon outflow in most cases. Meanwhile, carbon emissions, energy intensity, and structure had significant roles. Zhu et al. (2018) investigated the impacts of total demand, demand structure, Leontief structure, and emission efficiency on India's carbon emissions using additive SDA. Using the additive SDA, the authors calculated the effects of the total population, income per capita, expenditure/income ratio, demand structure, Leontief structure, and emission efficiency on household demand-embedded carbon emissions. Finally, they measured the effects of overall emissions intensity, emission intensity, Leontief structure, and demand structure on India's carbon intensity using the multiplicative SDA. According to the findings, private consumption was the primary driver of India's carbon emissions, followed by investment and exports. When it came to gauging India's relative emission efficiency, the aggregate embodied intensity indicator was driven mainly by private consumption, investment, and exports. Improvements in emission efficiency contributed little to lowering India's overall emissions and carbon intensity. Wang et al. (2017) estimated the global and national CO_2_ emission intensities' effects on emission intensity, production structure, demand structure, and geographic structure using the SDA technique. The findings indicated that sectoral emission efficiency increase was the primary factor contributing to the minor drop in global emission intensity. In contrast, international trade inhibited worldwide emission intensity improvements somewhat.

Besides energy, specific fossil fuels, and carbon emissions, the literature on related topics has examined the drivers of pollutant releases, water footprint, and land usage. Ščasný et al. (2021) measured the impacts of scale, fuel mix, structure, fuel intensity, and emission-fuel intensity on pollutant emissions in the Czech Republic by using the IDA approach. Emissions were reduced significantly in the 1990s, constant afterward, and decreased until 2019. Liu and Liang (2017) examined the impacts of structure and technology on various pollutants and carbon multipliers in China using the SDA. According to their results, emission multipliers were falling in general. The primary reason for this decline was technological advancements. Wang et al. (2016) analyzed the determinants of China's water footprint into technical, gross economic size, sectoral linkage, economic structure, and population using the SDA approach. The technical and economic structure impacts always outweighed the rise in water footprint, but the effect of gross economic size always harmed water use efficiency. Wang et al. (2021) investigated the causes of changes in embodied agricultural land usage in several regions of China using the SDA technique. Their SDA findings indicated that changes in embodied cropland might be related to characteristics such as cultivated land usage intensity and consumption level in most provinces.

Some studies have also considered the drivers of capital investment or manipulated capital investment as a production industry to understand its impact on the drivers of other final demand categories. Södersten et al. (2018a) assessed the influence of input–output multipliers and investment structure on the difference in the carbon intensity of capital investments using the SDA methodology. Their results indicated that the investment structure matters more for developing nations than industrialized ones. Cao et al. (2019a, b) used the SDA and capital formation as the production sector to estimate the effects of fossil fuel consumption structure, fossil fuel consumption per capita, carbon emission intensity, technology, consumption structure, per capita consumption, urbanization, and population on Chinese rural and urban residents' demand-embedded carbon emissions. The level of urbanization and total population, according to SDA findings, were both critical factors promoting carbon consumption by residents, with the former being more powerful. As per capita product consumption increased, so did the embedded carbon emissions of households, whereas the carbon emission intensity of industrial output decreased the embedded carbon emissions.

## Data sourcing and processing

The EORA MRIO database is used in this study to obtain China's national temporal IO tables from 1990 to 2016 (Lenzen et al. 2012, 2013). The Chinese IO data are divided into 122 sectors. To improve presentation and comprehension, we aggregated the EORA MRIO's 122 sectors into 13 major sectors. The reclassification details for the IO tables are shown in Table 1. The expenditure approach was used to estimate GDP, which adds up various final demand accounts to calculate a country's GDP value. The EORA MRIO database is also used to retrieve petroleum consumption information. Recent studies on carbon emissions (Sajid 2020; Sajid and Gonzalez 2021; Sajid et al. 2021b), energy use (Sajid et al. 2022), and other environmental issues (Sajid and Rahman 2021) have used the EORA MRIO as their primary data source.

**Table 1:** Sectoral classification from EORA MRIO and our study

Original sector from EORA MRIO	Our classification
"Crop cultivation; Forestry; Logging and transport of timber and bamboo; Livestock and livestock products; Fishery; Technical services for agriculture, forestry, livestock and fishing"	Agriculture
"Coal mining and processing; Crude petroleum products and Natural gas products; Ferrous ore mining; Non-ferrous ore mining; Salt mining; Non-metal minerals and other mining"	Mining and quarrying
"Grain mill products; Feeding stuff production and processing; Vegetable oil and forage; Sugar refining; Slaughtering, meat processing, eggs and dairy products; Prepared fish and seafood; Other food products; Wines, spirits and liquors; Non-alcoholic beverage; Tobacco products"	Food, beverages and tobacco
"Cotton textiles; Woolen textiles; Hemp textiles; Other textiles not elsewhere classified; Knitted mills; Wearing apparel; Leather, furs, down and related products"	Textile and clothing
"Sawmills and fiberboard; Furniture and products of wood, bamboo, cane, palm, straw, etc.; Paper and products; Printing and record medium reproduction; Cultural goods; Toys, sporting and athletic and recreation products; Arts and crafts products; Other manufacturing products"	Wood products and other manufacturing
"Petroleum refining; Coking; Raw chemical materials; Chemical fertilizers; Chemical pesticides; Chemicals for painting, dying and others; Synthetic chemicals; Chemicals for special usages; Chemical products for daily use; Medical and pharmaceutical products; Chemical fibers; Rubber products; Plastic products"	Fuel, chemical and plastic
"Cement and cement asbestos products; Glass and glass products; Pottery, china and earthenware; Fireproof products; Other non-metallic mineral products"	Non-metallic products
"Iron-smelting; Steel-smelting; Steel-processing; Alloy iron smelting; Nonferrous metal smelting; Nonferrous metal processing; Metal products"	Metal processing and products
"Boiler, engines and turbine; Metalworking machinery; Other general industrial machinery; Agriculture, forestry, animal husbandry and fishing machinery; Other special industrial equipment; Railroad transport equipment; Motor vehicles; Vehicles fittings production; Ship building; Other transport machinery; Generators; Household electric appliances; Other electric machinery and equipment; Communication equipment; Electronic computer; Other computer devices; Electronic element and device; Electronic appliances; Other electronic and communication equipment; Instruments, meters and other measuring equipment; Cultural and office equipment"	Machinery and equipment
"Scrap and waste; Electricity and steam production and supply; Gas production and supply; Water production and supply"	Utilities and waste services
"Construction"	Construction
"Railway passenger transport; Railway freight transport; Highway freight and passengers transport; Domestic public transport; Water freight and passengers transport; Air passenger transport; Air freight transport; Pipeline transport; Warehousing"	Transport and warehousing
"Post; Telecommunication; Computing services and software; Wholesale and retail trade; Hotels; Eating and drinking places; Finance; Insurance; Real estate; Leasehold; Business services; Tourism; Scientific research; General technical services; Geological prospecting; Water conservancy; Environmental resources and public infrastructure; Resident services and other services; Educational services; Health services; Social welfare; Culture and arts, radio, film and television; Sports; Recreational services; Public administration and other sectors"	Other services, recreation and entertainment

## Methods

### Environmentally extended input–output analysis

The following equation can present the typical Leontief demand model (Leontief 1936).

1 $$\begin{array}{*{20}c} {X = \left( {I - A} \right)^{ - 1} D = \left( {I - A} \right)^{ - 1} \left( {{\text{HS}} + {\text{GN}} + {\text{EX}} + {\text{GC}} + {\text{OT}}} \right)} \\ \end{array}$$

where $X$ presents the total sectoral output of a country or region. $A$ represents the technology matrix with element $a_{kl} = \frac{{x_{kl} }}{xl}$ presents the per unit input demand of sector $l$ for sector's goods or services. $L = \left( {I - A} \right)^{ - 1}$ is the matrix of Leontief's inverse. D is a matrix of final demand. ${\text{HS, GN, EX,GC,}}$ and ${\text{OT}}$ depict household, government, exports, gross fixed capital formation, and O present and other final demand categories, respectively. After estimating the A matrix based on total output, equation one is modified to only include the final demand category of ${\text{GC}}$.

2 $$\begin{array}{*{20}c} {X = \left( {I - A} \right)^{ - 1} {\text{GC}}} \\ \end{array}$$

Equation two can be extended to include the vector of sectoral petroleum intensity.

3 $$\begin{array}{*{20}c} {{\text{PGC}} = \hat{p}\left( {I - A} \right)^{ - 1} {\text{GC}}} \\ \end{array}$$

where ${\text{PGC }}$ presents the ${\text{GC}}$-induced vector of sectoral petroleum consumption. And $\hat{p}$ presents the diagonalized vector of sectoral petroleum intensity. The following equation depicts the procedure for estimating the vector of sectoral petroleum intensity $p$.

4 $$\begin{array}{*{20}c} {p = \mathop \sum \limits_{i = 1}^{n} \frac{{P_{i} }}{{x_{i} }}} \\ \end{array}$$

### SDA

The SDA and the IDA are two of the most common approaches to decomposing environmental impacts (Zhang et al. 2017b; Sajid 2021). SDA is recommended for dissecting the causes of indirect industrial ecological implications because it is more thorough in assessing the influence of final demand and technology, whereas IDA is used chiefly for estimating direct environmental (emissions) effects (Zhang et al. 2017b; Sajid 2021). The SDA is now a standard method for analyzing the impact of various factors on energy use, carbon emissions, and economic progress in the literature (Chen et al. 2019b). The SDA is a "comparative" statistical method that uses input–output-related data to measure economic structural changes. The SDA says that the overtime variations in a variable (like output, emissions, energy) are decomposed into the variation in its determining factors; the method is extensively employed to calculate the "underlying" causes of the variation (Dietzenbacher and Hoekstra 2002). In other words, the SDA splits the progression in a variable into the variations in its determining factors (Dietzenbacher and Los 1998). The currently used SDA typically decomposes the impact of different drivers on the industrial output, either physical or environmental, see (Dietzenbacher and Los 1998; Milana 2001; Dietzenbacher and Hoekstra 2002; Cao et al. 2019a; DOAN and Long 2019). The bipolar SDA is among the most popular type of SDA (Cao et al. 2019a; Sajid 2020, 2021). The bipolar SDA approach simplifies calculations and yields results that are more representative of the average of all possible decompositions (Nie et al. 2016).

To quantify the temporal impact of different factors, including the direct petroleum intensity $E = \hat{p}$, technology $T = \left( {I - A} \right)^{ - 1}$, capital investment structure $C = \frac{{{\text{GC}}_{i} }}{{{\text{GC}}}} \left( {i = 1,2,3 \cdots n} \right)$, investment intensity $I = \frac{{{\text{GC}}}}{{{\text{GDP}}}}$, and economic development $G = {\text{GDP}}$, equation number 3 can be decomposed in the following manner.

5 $$\begin{array}{*{20}c} {{\text{PGC}} = \mathop \sum \limits_{i = 1}^{n} \hat{p}\left( {I - A} \right)^{ - 1} \frac{{{\text{GC}}_{i} }}{{{\text{GC}}}} \times \frac{{{\text{GC}}}}{{{\text{GDP}}}} \times {\text{GDP}} = E \times T \times C \times I \times G} \\ \end{array}$$

If we present the base year by $b$ and the current year by $k,$ then the ${\text{GC}}$-stimulated petroleum consumption in the base and the recent years can be given as below:

6 $$\begin{array}{*{20}c} {{\text{PGC}}^{b} = E^{b} T^{b} C^{b} I^{b} L^{b} } \\ \end{array}$$

7 $$\begin{array}{*{20}c} {{\text{PGC}}^{k} = E^{k} T^{k} C^{k} I^{k} G^{k} } \\ \end{array}$$

where ${\text{PGC}}^{b}$ and ${\text{PGC}}^{k}$ represent the factor decomposition of ${\text{GC}}$-stimulated petroleum consumption in the base and the current years. The following equation presents the structural effect of changes between the base and the current year.

8 $$\begin{array}{*{20}c} {\Delta {\text{PGC}} = {\text{PGC}}^{k} - {\text{PGC}}^{b} = E^{k} T^{k} C^{k} I^{k} G^{k} - E^{b} T^{b} C^{b} I^{b} G^{b} } \\ \end{array}$$

The decomposition of Eq. 8 in the base year is presented as:

9 $$\begin{array}{*{20}c} {\Delta {\text{PGC}} = \underbrace {{\Delta ET^{b} C^{b} I^{b} G^{b} }}_{\Delta E} + \underbrace {{E^{K} \Delta TC^{b} I^{b} G^{b} }}_{\Delta T} + \underbrace {{E^{k} T^{k} \Delta CI^{b} G^{b} }}_{\Delta C} + \underbrace {{E^{k} T^{k} C^{k} \Delta IG^{b} }}_{\Delta I} + \underbrace {{E^{k} T^{k} C^{k} I^{k} \Delta G}}_{\Delta G}} \\ \end{array}$$

The decomposition of Eq. 8 in the current year is presented as:

10 $$\begin{array}{*{20}c} {\Delta {\text{PGC}} = \underbrace {{\Delta ET^{k} C^{k} I^{k} G^{k} }}_{\Delta E} + \underbrace {{E^{b} \Delta TC^{k} I^{k} G^{k} }}_{\Delta T} + \underbrace {{E^{b} T^{b} \Delta CI^{k} G^{k} }}_{\Delta C} + \underbrace {{E^{b} T^{b} C^{b} \Delta IG^{k} }}_{\Delta I} + \underbrace {{E^{b} T^{b} C^{b} I^{b} \Delta G}}_{\Delta G}} \\ \end{array}$$

By taking the average of Eqs. 9 and 10, the bipolar decomposition equations representing the impact of different factors can be presented as below:

11 $$\begin{array}{*{20}c} {\Delta E = \frac{1}{2}\left( {\Delta ET^{b} C^{b} I^{b} L^{b} + \Delta ET^{k} C^{k} I^{k} G^{k} } \right)} \\ \end{array}$$

12 $$\begin{array}{*{20}c} {\Delta T = \frac{1}{2}\left( {E^{K} \Delta TC^{b} I^{b} L^{b} + E^{b} \Delta TC^{k} I^{k} G^{k} } \right)} \\ \end{array}$$

13 $$\begin{array}{*{20}c} {\Delta C = \frac{1}{2}\left( {E^{k} T^{k} \Delta CI^{b} L^{b} + E^{b} T^{b} \Delta CI^{k} G^{k} } \right)} \\ \end{array}$$

14 $$\begin{array}{*{20}c} {\Delta I = \frac{1}{2}\left( {E^{k} T^{k} C^{k} \Delta IL^{b} + E^{b} T^{b} C^{b} \Delta IG^{k} } \right)} \\ \end{array}$$

15 $$\begin{array}{*{20}c} {\Delta G = \frac{1}{2}\left( {E^{k} T^{k} C^{k} I^{k} \Delta L + E^{b} T^{b} C^{b} I^{b} \Delta G} \right)} \\ \end{array}$$

Similarly, the equation representing the combined impact of different factors can be rewritten as:

16 $$\begin{array}{*{20}c} {\Delta PGC = \Delta E + \Delta T + \Delta C + \Delta I + \Delta L} \\ \end{array}$$

## Results

### China's temporal IEPC

**Fig. 1:**
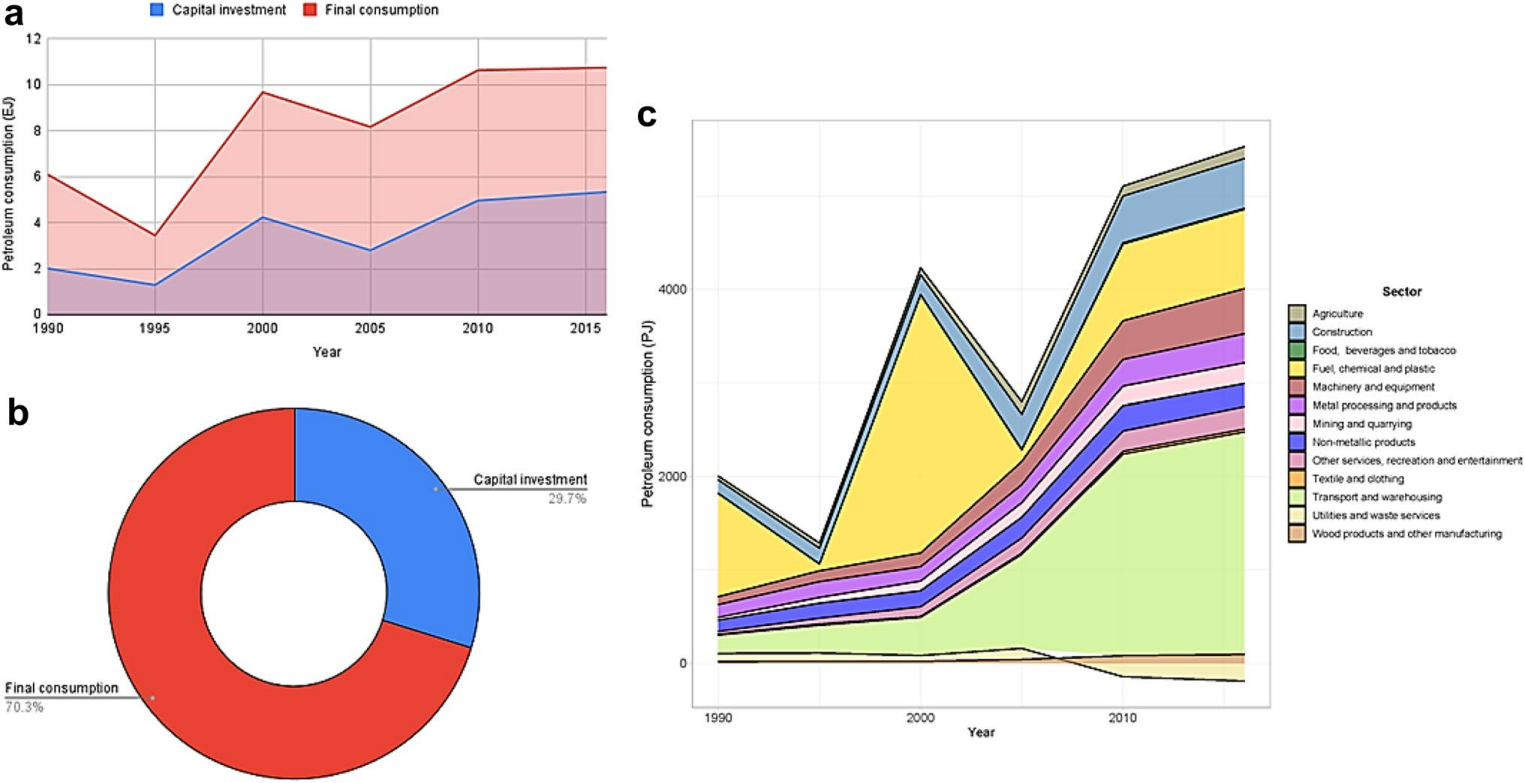
China's temporal IEPC. **a** Temporal final demand-embedded petroleum consumption. **b** Average final demand-embedded petroleum consumption from the year 1995 to 2016. **c** Sector-wise temporal IEPC

Figure 1 depicts China's temporal IEPC. Figure 1a shows that from 1990 to 2016, China's final demand embedded petroleum consumption, and its main components of investment and final consumption increased significantly. However, a significant disparity existed in the growth of final demand-embedded petroleum consumption in China's investment and consumption over the years. Between 1990 and 2016, China's IEPC grew at a compound annual growth rate (CAGR) of 17.73%, while final consumption-embedded petroleum consumption grew at a CAGR of only 9.91%. Figure 1b shows that, despite the larger disparity in the growth rate of embedded petroleum usage between investment and final consumption, nearly 70% of petroleum consumption was embedded in final consumption. Figure 1c depicts the IEPC by sector. Approximately 1103 and 2760 petajoule (PJ) of petroleum use was embedded in Chinese investment in the fuel, chemical, and plastic sectors between 1990 and 2000. Transport and warehousing had the highest ever-increasing IEPC in 1995 (297 PJ) and from 2005 onward.

### SDA of factors temporal impacts

Table 2 shows the yearly impacts of various factors on China's IEPC. Between 1990 and 1995, the Chinese IEPC fell by − 0.72 exajoules (EJ). Petroleum use intensity, at − 2.61 EJ, was primarily responsible for this decrease in China's IEPC. During this time, all other factors had a positive driving impact on the IEPC in China. However, in absolute terms, this positive impact was less than the negative impact of petroleum intensity, resulting in an overall decrease in IEPC. Except for the investment structure (− 0.03 EJ), all factors increased the IEPC between 1995 and 2000. Petroleum intensity contributed the most to this increase, with a value of 0.26 EJ. The IEPC decreased by − 1.43 EJ between 2000 and 2005, primarily due to the petroleum intensity (− 4.38 EJ). The factor of economic development had the chief upward impact during this period (1.78 EJ). Between 2005–2010 and 2010–2016, China's IEPC increased by 2.16 EJ and 0.38 EJ, respectively. During this time, petroleum intensity had the greatest downward pulling impact on IEPC, with − 2.81 EJ and − 2.13 EJ values. In contrast, investment intensity (2.09 EJ and 1.80 EJ) and economic development (2.02 EJ and 2.36 EJ) exerted the most significant upward pressure on the IEPC. From 1990 to 2016, the average petroleum intensity of − 2.19 EJ had the highest negative impact on IEPC. On average, both investment intensity (1.09 EJ) and economic development (1.66 EJ) had the biggest positive impact on IEPC. Compared to other factors, the average effects of remaining technology (0.15 EJ) and investment structure (− 0.04 EJ) were relatively minor.

**Table 2:** Temporal impact of different factors on China’s IEPC

Item	1990**–**1995	1995**–**2000	2000**–**2005	2005**–**2010	2010**–**2016	Average impact
Petroleum intensity	− 2.61	0.97	− 4.38	− 2.81	− 2.13	− 2.19
Technology	0.68	0.71	0.29	0.87	− 1.51	0.15
Investment structure	0.02	− 0.03	− 0.03	− 0.001	− 0.15	− 0.04
Investment intensity	0.42	0.26	0.91	2.09	1.80	1.09
Economic development	1.07	1.05	1.78	2.02	2.36	1.66
Aggregate impact	− 0.72	2.94	− 1.43	2.16	0.38	–

^*^Unit = EJ

### Factors impacts on sector-wise IEPC

**Fig. 2:**
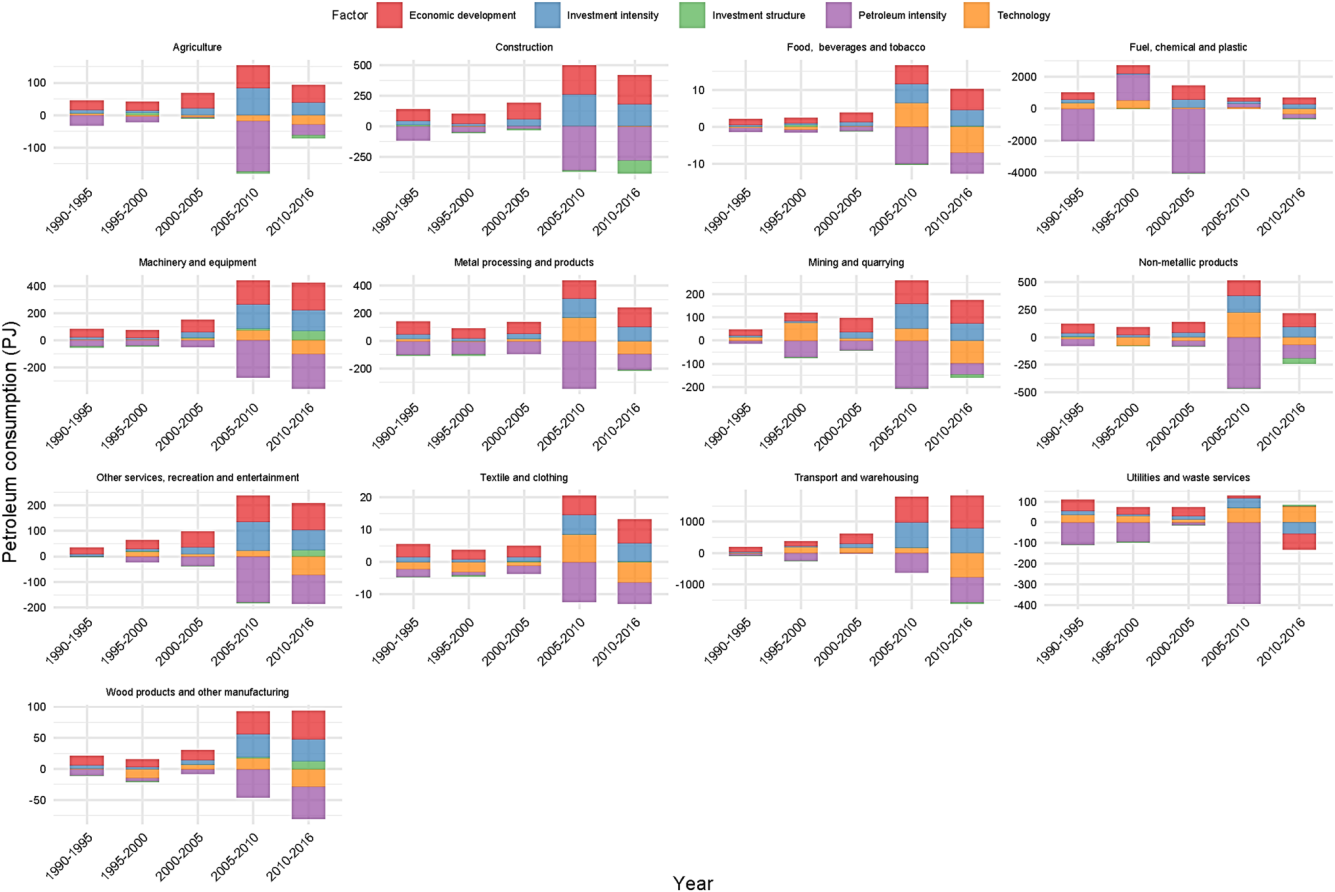
The temporal impacts of different factors on China's sectoral IEPC

Figure 2 depicts the temporal effects of various factors on China's sectoral IEPC. Figure 2 shows that the Chinese sectoral IEPC had different temporal effects from similar factors. Agriculture; Metal processing and products; Mining and quarrying; Non-metallic products; Utilities and waste services, and the Textile and clothing sectors, for example, had significantly larger absolute (positive and negative) effects from 2005 to 2010. Construction; Food, beverages, and tobacco; Machinery and equipment; Other services, recreation, and entertainment all had significant absolute effects in 2005–2010 and 2010–2016. Between 2010 and 2016, Transportation and warehousing; Wood products and other manufacturing had a significantly larger socioeconomic factor impact on their respective IEPCs. The IEPC of the Fuel, chemical, and plastics sector was the most impacted by the selected factors between 2000 and 2005.

Table 3 presents the average impact of different factors on sectoral IEPC from 1995 to 2016. Across all sectors, the petroleum intensity had the highest negative impact on IEPC. The petroleum intensity had the greatest impact on the sectors of Fuel, chemical, and plastic (− 895.01 PJ); Transportation and warehousing (− 355.83 PJ); and Construction (− 165.60 PJ). In contrast, the effects of technology and investment structure on IEPCs from various sectors were mixed. However, the average impact of technology on sectoral IEPCs in absolute value terms was significantly larger than the investment structure, while both the investment intensity and economic development positively impacted the sectoral IEPC. In general, economic development had a bigger impact than investment intensity. Investment intensity and economic development had the greatest impact on Transportation and warehousing (359.98 PJ and 488.06 PJ); Fuel, chemical, and plastic (250.65 PJ and 475.43 PJ); and Construction (111.31 PJ and 155.81 PJ) sectors IEPCs.

**Table 3:** The average impact of different factors on sectoral IEPC from 1995 to 2016

Item	Petroleum intensity	Technology	Investment structure	Investment intensity	Economic development
Agriculture	− 48.28	− 10.01	− 1.88	32.43	44.91
Mining and quarrying	− 77.21	10.65	− 3.27	45.53	62.85
Food, beverages, and tobacco	− 3.75	− 0.22	0.10	2.30	3.20
Textile and clothing	− 4.92	− 0.89	− 0.03	3.09	4.71
Wood products and other manufacturing	− 24.14	− 3.58	2.72	17.49	25.19
Fuel, chemical, and plastic	− 895.01	132.16	− 14.04	250.65	475.43
Non-metallic products	− 142.00	8.46	− 9.58	67.78	100.42
Metal processing and products	− 150.03	19.24	− 2.80	66.64	101.54
Machinery and equipment	− 133.20	− 0.35	15.01	82.10	116.02
Utilities and waste services	− 121.48	44.50	0.70	7.93	12.57
Construction	− 165.60	− 0.36	− 23.12	111.31	155.81
Transport and warehousing	− 355.83	− 48.56	− 6.70	359.98	488.06
Other services, recreation, and entertainment	− 70.81	− 4.52	4.76	47.16	65.08

^*^Unit = PJ

## Discussion

Petroleum is the most widely used fossil fuel on a global scale. Capital investment is a key indicator of long-term economic development. Unlike consumption, which has a short-term impact on GDP, capital investments have a long-term impact on regional or national GDP. Investment and petroleum, in particular, play critical roles in the economic development of rapidly developing economies. However, few studies have estimated the IEPC drivers. We used China as a case study because it is one of the world's largest consumers and importers of petroleum. At the same time, the country leads the world in capital investment. Moreover, despite the COVID-19 pandemic in 2020, China witnessed an increase of 2.2% in fossil fuel consumption over the previous five years (U.S. Energy Information Administration’s (EIA) 2021; Sajid et al. 2022). Other GDP items, such as investment, may be important in simulating Chinese industrial sectoral fossil fuel consumption and driving the impact of final consumption (which was affected to some extent by COVID-19-related lockdowns). Understanding the main socioeconomic factors that affect IEPC could help China and other countries cut back on fossil fuel use. Using the well-known bipolar SDA, we estimated the socioeconomic drivers of Chinese IEPC from 1990 to 2016.

The findings revealed that capital investment embedded a significant portion of Chinese petroleum consumption. In general, the IEPC share increased faster than the final demand-embedded petroleum consumption share. According to final demand-embedded fossil fuel/energy consumption studies, there are different sector-wise shares of embedded energy consumption patterns in China over different periods (Chen et al. 2019a). For example, the Construction and Chemical industries consumed the most energy in China between 1993 and 1997, Construction and Services between 1998 and 2002, and Metal smelting and Construction between 2003 and 2007 (Sun et al. 2016). Furthermore, Sajid et al. (2022) demonstrated that in 2016, the Petroleum refining and Transportation-related sectors had the highest final demand-embedded petroleum consumption in China. Our findings also revealed dynamic patterns of sectoral contributions to IEPC over time. Between 1990 and 2000, the largest IEPC came from the Fuel, chemical, and plastic sector. Transport and warehousing had the highest IEPC between 1995 and 2005. However, when compared to previous findings related to the final demand-embedded fossil fuel and petroleum consumptions, our findings are primarily related to the IEPC. That underscores the importance of investments across various sectors in terms of indirect petroleum consumption. Besides, the results serve as the foundation for the estimation of the effects of various socioeconomic factors on the Chinese IEPC.

Several studies have discovered that capital investment is critical in determining the total value of final demand-embedded fossil fuel/energy consumption. For example, Li et al. (2020) found that between 2012 and 2017, capital investment was the main increasing factor in China's embedded energy intensity. According to Yan and Su (2020) and Zhang et al. (2017a), capital investment is the most important driving factor in China's final demand-embedded fossil fuel/energy consumption. Despite these findings, few studies have looked into the factors of investment-embedded energy impacts, particularly on petroleum consumption. To the best of the authors' knowledge, the solo work was conducted by Södersten et al. (2018a); they investigated the effects of input–output multipliers and investment structure on the carbon intensity of capital investments across different states. However, as is evident, this work did not investigate the IEPC, and only two factors, namely the input–output multiplier and the investment structure, were investigated. Our findings differ from previous findings on the factors of embedded environmental impacts on three main grounds: (1) In general, the socioeconomic factors of IEPC have not been studied. (2) Studies on the factors of investment-embedded environmental impacts investigate only one or two factors (see, for example, Södersten et al. (2018a)). (3) Furthermore, the investment intensity factor (index) introduced in our study is not commonly used in final demand-embedded environmental impacts factor studies (the literature review section provides a thorough analysis of factors commonly investigated by related works).

According to our bipolar SDA, the petroleum intensity had the highest negative impact on the Chinese IEPC. Except for 2010–2016, technology had a net positive impact on the Chinese IEPC. Over the 1990–2016 study period, investment intensity also positively impacted China's IEPC. For the years 1990–1995 and 1995–2000, the positive impact of technology on Chinese IEPC was slightly greater than investment intensity. From 2000 to 2016, the absolute effect of investment intensity outweighed the effects of technology. During the period 2010–2016, sectoral technology (per unit input (material) requirements) improved significantly, and the technology had a decreasing impact on IEPC of nearly − 1.51 EJ. This demonstrates that, following a period of poor performance, China's sectoral technology improved significantly between 2010 and 2016. During the study period, economic development (GDP) had the greatest positive impact on China's IEPC. On average, investment structure had the least negative impact on China's IEPC. Other studies, such as Liu et al. (2019), Li et al. (2020), Yan and Su (2020), and Huang et al. (2022), discovered that energy intensity played a significant role in lowering the final demand-embedded fossil fuel/energy consumption in China and its various regions. Andreoni (2022) also demonstrated that China's coal intensity has a negative effect on regional coal consumption. Several studies have also found that economic development (prosperity) has a significant positive impact on the final demand-embedded fossil fuel/energy consumption. According to Liu et al. (2019), economic development played an important role in increasing the final demand-embedded fossil fuel/energy consumption in China. Andreoni's (2022) findings also revealed that economic development was the primary driver of China's rising coal consumption. Furthermore, studies such as Yan and Su (2020) and Liu et al. (2019) found that the technology (Leontief effect) had an overall negative impact on final demand-embedded fossil fuel/energy consumption. In addition, Liu et al. (2019) demonstrated that consumption structure had little impact on final demand-embedded fossil fuel/energy consumption. After discussing the findings of several recent studies that support our results, it should be noted that our findings are novel in comparison with these supporting studies. Previous works have generally focused on final demand rather than capital investment. Second, none of the studies discussed above or in the literature review sections have considered the investment intensity factor proposed in our research.

The disaggregation of the effects of various factors at the sectoral level yielded some intriguing results. The petroleum intensity, which, as previously stated, had the chief decreasing impact on China's IEPC on average, had the biggest negative effects on IEPCs in the Fuel, chemical, and plastic; Construction; Transportation and warehousing sectors. These three sectors also had the largest positive impacts on their respective IEPCs from investment intensity and economic development factors. On average, the sectors of Fuel, chemical, and plastic, as well as utilities and waste services, had the greatest positive impact of technology on their IEPCs. In contrast, technology had the greatest average negative impact on the IEPC of the Transport and warehousing sector. It implies that, on average, technology or, more specifically, material consumption efficiency in these two sectors did not improve but instead deteriorated. While technology in China's Utilities and waste services sector improved dramatically, it had the greatest negative impact on China's IEPC. Finally, the investment structure had the greatest negative impact on the IEPC in the sector of Fuel, chemical, plastic, and Construction sectors. In contrast, it had the greatest positive impact on the IEPC of Machinery and equipment.

## Policy implications

The current COVID-19 lockdowns and economic slowdown have slowed global energy consumption growth and associated carbon emissions. Given the rapid economic recovery in major Asia–Pacific countries, including China, it has been argued that COVID-19 demand shocks will harm annual energy-related CO_2_ emissions by less than 1% in the coming years (Sajid and Gonzalez 2021). Thus, major global economies such as China must avoid relying on short-term relief from energy consumption caused by COVID-19-related economic activity disruptions and instead focus on long-term energy consumption mitigation for critical energy resources such as petroleum. The IEPC is one such unexplored frontier. The IEPC can be incorporated into long-term energy policies to generate economic and environmental benefits for major petroleum consumers and import-dependent, large capital investment-intensive, fast-growing economies like China. The following section provides policy recommendations based on our findings that may help guide China's and other countries' environmentally sustainable development.

As per our findings, the capital investment embedded a significant portion of China's petroleum consumption. On average, factors such as petroleum intensity, technology (between 2010 and 2016, the most recent period considered), and investment structure had a negative impact on China's IEPC. As a result, further improvements in petroleum intensity through fuel-efficient machinery and labor training, technological innovations, and improvements in investment structure can amplify the decreasing effects of these factors and aid in reducing China's IEPC total volume.

At the sectoral level, the sectors of Fuel, chemical, and plastic; and Utilities and waste services, on average, showed a significant diminishing trend of technological improvements, increasing the effect of technology in escalating IEPC. In comparison, the Transportation and warehousing sector demonstrated the greatest technological advancements in terms of negative impacts on IEPC. As a result, significant investments in technological advancements in these three sectors can help boost overall IEPC reductions. The negative effects of the investment structure can be exacerbated further by conditioning investments in sectors such as Machinery and equipment to subsequent improvements in petroleum intensity and technology.

Both the investment intensity and the rate of economic development have had a significant increasing effect on China's IEPC. It is well understood that investment is critical for long-term economic growth. As a result, investment cannot be easily separated from economic development. The challenge for both industrial environmental managers and government policymakers is to achieve environmentally sustainable investment, in this case, concerning petroleum embedded investment. The long-run wealth generated by increased investment share (increased investment intensity) can be reinvested in acquiring advanced technology (petroleum discovery, drilling, production, and the consuming sectors machinery and equipment, for example) and in research and development of new technologies and techniques. The second option is to redirect current petroleum demand to alternative and renewable options (A&R) via investments in A&R.

### Theoretical and managerial implications

Our study quantifies the effects of several socioeconomic factors associated with IEPC that have been overlooked in previous research, thus making a significant theoretical contribution. Our work's methodological contribution, in particular, can assist in evaluating the impact of various socioeconomic factors on the environmental effects of investments (fixed asset development). Thus, it can help with the targeted and effective mitigation of the environmental impact of fixed asset formation. Additionally, the inclusion of a novel indicator such as the index of investment intensity, which estimates investment growth concerning the GDP, adds to our study's theoretical novelty.

Our research has several managerial-specific policy implications besides the general (government and managerial) policy and theoretical implications discussed above.

Prior research indicates that macro-environmental and socioeconomic indicators are related to green management practices (Khan et al. 2021e, b). Additionally, it is demonstrated that environmental and economic sustainability can significantly improve an organization's performance (Khan et al. 2020, 2021a). Economic and environmental benefits can be realized by investing in environmentally responsible infrastructures (fixed assets) (Khan et al. 2021c). The findings of our study can assist industrial managers, particularly environmental managers and consultants, in making environmentally responsible fixed investments. That is, by understanding the IEPC of specific assets, particularly for managers in major petroleum-importing developing economies, the acquisition or development of fixed assets can be shifted toward less IEPC-intensive alternatives.

Furthermore, previous evidence has indicated that, in general, both carbon emissions and taxes can be reduced through technological advancements and innovations (Khan et al. 2021d). The analysis of major influencing factors such as petroleum intensity and technology in our study can assist managers in concentrating their efforts on more efficient fossil fuel production technologies. Furthermore, intensity improvements, in addition to technological advancements, would necessitate and encourage the use of innovations such as carbon capture and storage, labor training (such as controlling oil spills, wastage control via switching off idle machinery and equipment, and so on), and the use of alternative and renewable energy to the greatest extent possible. This can help not only with improved corporate social responsibility standards but also with lower carbon footprints.

### Limitations and future research directions

Our study concentrated on China's IEPC's understudied drivers. Future work may extend and modify the bipolar SDA-based factor impacts covered in our work to include other fossil fuels, alternative energy sources, and various environmental effects. In particular, the novel investment intensity index proposed in our work may be applied in future research.

## Data Availability

The input–output and petroleum consumption data can be found on the EORA MRIO website (https://worldmrio.com/). Additionally, data on China's IEPC and the effects of socioeconomic factors on IEPC are included in the manuscript's tables and figures.

## Footnotes

1 Capital Formation is classified into three types: Gross Fixed Capital Formation (GFCF), Inventory Changes, and Acquisitions (less disposals) of valuables (Central Statistics Office 2021). GFCF, also known as "investment," is described as the purchase of manufactured assets (including used assets) and the development of such assets by producers for their own use, minus disposals (OECD 2022). The pertinent assets are those that are likely to be employed in the production of other goods and services for a period of time longer than one year (OECD 2022).

2 Over the next several decades, the Chinese petroleum sector is expected to gradually increase to a share of about 20% of the total energy mix (Yang et al. 2016). Although coal accounted for 57.64% of China's energy consumption in 2019, petroleum accounted for 19.69% (Ritchie and Roser 2020c). In contrast to petroleum, China is the world's largest producer of coal (bp (British Petroleum) 2021). Chinese coal production was only 2.98% less than consumption in 2019 [estimations based on (Ritchie and Roser 2020d, e)]. Furthermore, China's discovered coal reserves can support its coal consumption for the next 35 years without requiring any imports (Worldometer 2016b). This demonstrates that, in comparison with petroleum, China's coal energy sources are relatively secure.

3 GDP is the broadest and most often used measure of national income (Ott 2019).

## Code availability

The MATLAB code for China's IEPC and bipolar SDA is available upon reasonable request from the corresponding author.
